# Heparin Interaction with the Primed Polymorphonuclear Leukocyte CD11b Induces Apoptosis and Prevents Cell Activation

**DOI:** 10.1155/2015/751014

**Published:** 2015-12-27

**Authors:** Meital Cohen-Mazor, Rafi Mazor, Batya Kristal, Erik B. Kistler, Inbal Ziv, Judith Chezar, Shifra Sela

**Affiliations:** ^1^Eliachar Research Laboratory, Western Galilee Hospital, 22100 Nahariya, Israel; ^2^Technion Faculty of Medicine, 3525433 Haifa, Israel; ^3^Department of Nephrology and Hypertension, Western Galilee Hospital, 22100 Nahariya, Israel; ^4^Department of Anesthesiology & Critical Care, VA San Diego Healthcare System, San Diego, CA 92161, USA; ^5^Hematology Laboratory, Western Galilee Hospital, 22100 Nahariya, Israel

## Abstract

Heparin is known to have anti-inflammatory effects, yet the mechanisms are not completely understood. In this study, we tested the hypothesis that heparin has a direct effect on activated polymorphonuclear leukocytes (PMNLs), changing their activation state, and can explain its anti-inflammatory effect. To test our hypothesis, we designed both* in vitro* and* ex vivo* studies to elucidate the mechanism by which heparin modulates PMNL functions and therefore the inflammatory response. We specifically tested the hypothesis that priming of PMNLs renders them more susceptible to heparin. Amplified levels of CD11b and increased rate of superoxide release manifested PMNL priming. Increase in cell priming resulted in a dose-dependent increase in heparin binding to PMNLs followed by augmented apoptosis. Blocking antibodies to CD11b inhibited heparin binding and abolished the apoptotic response. Moreover, heparin caused a significant dose-dependent decrease in the rate of superoxide release from PMNLs, which was blunted by blocking antibodies to CD11b. Altogether, this study shows that the interaction of heparin with the PMNL CD11b results in cell apoptosis and explains heparin's anti-inflammatory effects.

## 1. Introduction

In many inflammatory responses, polymorphonuclear leukocytes (PMNLs) are among the first cells to exit the blood stream and migrate to an inflammatory site, where they become fully activated. This activation is a two-stage process: PMNLs first encounter a stimulus that does not activate the cells directly but leaves them in a “primed” state. Then, upon encountering a second stimulus in the inflamed site, the transition into a fully activated state will occur [1, 2]. This process involves the production of free radicals and release of granule enzymes into the surrounding milieu. Therefore, tight regulation of PMNL activation is needed throughout the steps of infiltration from the blood stream to the inflamed site in order to prevent damage to the vascular wall and the extracellular matrix (ECM).

One of the ECM components is heparin (in the form of heparan sulfate), a soluble molecule that plays a major role in defining the physical and chemical properties of the ECM [3]. Heparin, which is commonly used as a blood anticoagulant, is also known to have anti-inflammatory effects; however, the mechanism of these biological activities remains largely unknown [4, 5]. Some of heparin's anti-inflammatory effects are mediated through the modulation of cellular activation, particularly of PMNLs [6–9]. Heparin decreases phorbol myristate acetate (PMA),* N*-formyl-methionyl-leucyl-phenylalanine (fMLP), and opsonized zymosan-induced superoxide production [7], a decrease which is even greater when the PMNLs are primed by platelet activating factor (PAF) [9]. Heparin reduces fMLP-stimulated PMNL adhesion to endothelial cells and decreases the release of beta-glucuronidase and lysozyme from stimulated PMNLs [6]. In addition, heparin has been shown to inhibit leukocyte recruitment and chemotaxis in response to zymosan-activated serum [10].

Recently, it was shown that immobilized heparin can mediate cell adhesion via interaction with the PMNL integrin Mac-1 (CD11b/CD18, *α*_M_ *β*_2_) [11]. Mac-1 is one of the most versatile adhesion molecules, with ligands of various biological functions. One of these functions is the induction of a signal transduction cascade that substantially augments apoptosis of activated PMNLs [12].

The above data, especially the known apoptotic effect of heparin on PMNLs [13, 14], led us to hypothesize that priming of PMNLs renders them more susceptible to the apoptotic effects of heparin and that apoptosis induced by heparin is mediated in part by heparin interactions with CD11b, which is highly expressed on the surface of primed PMNLs [15]. In order to test our hypothesis, we used PMNLs isolated from hemodialysis (HD) patients as a model of* in vivo* primed cells [16] and PMNLs isolated from healthy controls (NC) primed* ex vivo* with PAF. Our results indicate that primed PMNLs, regardless of their priming origin (*ex vivo* or* in vivo*), are more susceptible to the apoptotic effect of heparin compared to nonprimed PMNLs. We also show that heparin binds to CD11b, leading to apoptosis that can be blocked with neutralizing antibodies against CD11b.

## 2. Methods

### 2.1. Patients and Blood Samples

Blood was drawn from 17 patients on chronic hemodialysis and 24 age- and gender-matched healthy control subjects (NC). Blood for the determination of biochemical and hematological parameters and for the isolation of PMNLs was drawn after an overnight fast. Blood was collected into citrate tubes from the arterial line of all the HD patients immediately before a dialysis session. All patients underwent hemodialysis three times a week; each dialysis treatment lasted 4 hours and was carried out with low flux polysulfone membranes (F8, Fresenius Medical Care, Bad Homburg, Germany). The water for dialysis met the standards of the Association for the Advancement of Medical Instrumentation (AAMI). Patients with evidence of acute or chronic infection or malignancy or who had received blood transfusion within 3 months prior to blood sampling were excluded. All participants signed an informed consent for blood sampling, and the study was approved by the Institutional Committee in accordance with the Helsinki declaration.

### 2.2. PMNL Isolation and Analysis

PMNLs were isolated as described previously [17]. Isolated PMNLs (>98% pure, approximately 10^7^ cells per isolation) were resuspended and counted in phosphate buffered saline (PBS, Beit Haemek, Israel) containing 0.1% glucose. PMNL priming was assessed by the rate of superoxide release [17] and by the surface levels of CD11b, as described previously [15]. The rate of superoxide release was determined after cell stimulation with 0.32 × 10^−7 ^M phorbol 12-myristate 13-acetate (PMA; Sigma, St. Louis, MO). The assay is based on superoxide dismutase (SOD) inhibitable reduction of 80 *μ*M cytochrome C (Sigma, St. Louis, MO) to its ferrous form. The change in optical density was monitored at 549 nm, as described previously [17]. Expression of CD11b on PMNLs in whole blood was determined using the FC500 flow cytometer (Beckman-Coulter). 50 *μ*L of blood was incubated for 10 min with anti-CD11b-PE conjugated monoclonal antibody (Immunotech, Marseille, France), followed by red blood cell lysis (Q-prep method; Coulter Corporation, Hialeah, FL, USA). To enable gating on the PMNL population, anti-CD16, conjugated to PC5 monoclonal antibody (IQ Products), was used. Surface levels of CD11b on PMNLs are expressed as mean fluorescence intensity (MFI), after subtracting the nonspecific background.

### 2.3. Priming of PMNLs by Platelet Activating Factor (PAF)

PAF (Sigma, USA), a known dose-dependent priming agent of PMNLs [18], was used for the* in vitro* priming of NC PMNLs in three concentrations: 1 pM, 1 nM, and 1 *μ*M. Cells were incubated with PAF for 15 minutes at room temperature and then washed with PBS. In PAF-stimulated PMNLs where CD11b intensity was measured and in experiments where CD11b was blocked, 5 minutes after the initiation of incubation with PAF, mouse anti-human-CD11b-PE was added. CD11b expression was measured as described above for whole blood, but without the lysis step.

### 2.4. Effect of Heparin on PMNLs

The effect of heparin was studied on three types of isolated PMNLs: normal control (NC) PMNLs, PAF-stimulated NC PMNLs, and HD PMNLs. All cells were incubated for 30 min at room temperature with 25, 50, and 100 U/mL sodium heparin (Kamada, Beit Kama, Israel) diluted in PBS. In order to measure time-dependent effects of heparin, NC and HD PMNLs were incubated with 100 U/mL sodium heparin for 300 min at room temperature.

### 2.5. Analysis of PMNL Apoptosis

The percentage of apoptotic PMNLs was based on Annexin-V-FITC binding according to the manufacturer's recommendations (Annexin-V/FITC Kit, Bender MedSystems Diagnostics GmbH, Vienna, Austria). Annexin-V-FITC and propidium iodide (PI) were added to the cell suspension and incubated for 10 min in the dark at room temperature. PI staining is a dye-exclusion assay that discriminates between cells with intact membranes (PI negative) and permeabilized membranes (PI positive) [19]. The cell population showing Annexin-V−/PI− was considered viable; Annexin-V+/PI− was considered as an early apoptotic population. Late stage apoptotic or necrotic cells were represented by the Annexin-V+/PI+ population [19].

### 2.6. Analysis of PMNL Apoptosis after Preincubation with Anti-CD11b

Separated PMNLs (5 × 10^5^) were incubated with anti-CD11b-PE or its isotype control- (IC-) PE for 10 minutes, washed, and incubated for 30 minutes with 25 U/mL heparin. This dose of heparin was chosen since it is the minimal dose that caused a statistically significant difference in apoptosis between primed and nonprimed PMNLs. Apoptosis in PMNLs was detected using the following: (A) Annexin-V-FITC binding was measured as mentioned above without the addition of PI. The percentage of apoptotic cells was compared with apoptosis detected using FITC-labeled Annexin-V, added after preincubation with IC-PE (Immunotech, Marseille, France). The positive cutoff point was determined by FITC-isotype controls (IQ Products, Netherlands), added after preincubation with IC-PE. (B) PMNLs (5 × 10^6^) were cytospinned and apoptosis was detected with an Axioscop 2 upright fluorescence microscope, using the* In Situ* Cell Death detection kit (TUNEL staining, Roche Molecular Biochemicals). Nuclear staining was done with 5 *μ*g/mL Hoechst (Calbiochem).

### 2.7. PMNL Viability

Cells (2 × 10^6^) were incubated at room temperature for 0, 30, and 90 minutes in PBS with and without heparin (100 U/mL), stained with trypan blue solution (0.4% in HBSS), and counted.

### 2.8. Heparin Binding to PMNLs

Separated PMNLs (5 × 10^5^) were incubated with anti-CD11b-PE or its isotype control for 10 minutes, washed, and assayed for heparin binding by two different methods: (A) the PMNLs were further incubated with 25 U/mL sodium heparin. The percentage of PMNLs that bound heparin and the intensity of heparin binding to PMNLs were determined by flow cytometry using mouse anti-human heparan sulfate-FITC (United States Biological, Massachusetts). The Ab was generated by immunization with liposome-intercalated membrane HS proteoglycans and recognizes an epitope present in many types of human heparan sulfate including heparin. The epitope includes N-sulfated glucosamine residues that are critical for the reactivity of the antibody. The Ab does not react with hyaluronan, chondroitin sulfate, dermatan sulfate, keratan sulfate, or DNA [20, 21]. The positive cutoff point was determined by FITC-isotype control (IQ Products, Netherlands), added after preincubation with anti-CD11b-PE. The percentage of heparin bound cells and the intensity of heparin binding to PMNLs detected in this experiment were compared with cells detected using FITC-labeled anti-heparan sulfate, added after preincubation with IC-PE (Immunotech, Marseille, France). The positive cutoff point was determined by FITC-isotype controls (IQ Products, Netherlands), added after preincubation with IC-PE. (B) The PMNLs were further incubated with 25 U/mL FITC-labeled heparin (Molecular Probes, Eugene, OR) in PBS for 30 minutes at room temperature. The intensity of heparin binding to PMNLs was determined by FC500 flow cytometer (Beckman-Coulter).

### 2.9. Statistical Analysis

Data are expressed as mean ± SD. In the box and whiskers presentations, the horizontal line in the middle shows the median (50th percentile), the top and bottom of the box indicate the 75th and 25th percentiles, respectively, and the whiskers show the maximum and the minimum values. The nonparametric Mann-Whitney test was used for comparing two independent groups. The two-paired Wilcoxon Signed Ranks test was used for comparing two dependent groups. Statistical significance was considered at *P* < 0.05.

## 3. Results

### 3.1. PMNL Priming

**Figure 1:**
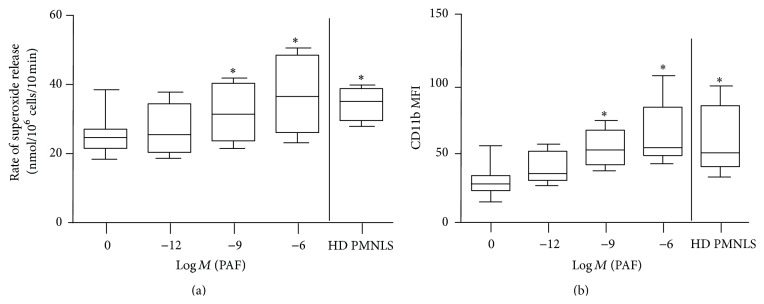
PMNL priming. (a) Rates of superoxide release from separated NC PMNLs after 15 min of stimulation by 1 pM, 1 nM, and 1 *μ*M PAF and HD PMNLs activated with 0.32 × 10^−7 ^M PMA. The changes in optical density were monitored at 549 nm continuously up to 10 min in the presence of 0.08 mM cytochrome C. Data are expressed as nmoles/10^6^ cells/10 min; ^*∗*^ *P* < 0.05 for HD and PAF stimulated (10^−9 ^M and 10^−6 ^M) versus nonstimulated NC PMNLs (no PAF), *n* = 10. (b) Relative expression of surface CD11b on PMNLs measured by flow cytometry using specific PE-labeled antibody, as described in Section 2. Data are expressed as mean fluorescent intensity (MFI); ^*∗*^ *P* < 0.05 for HD and PAF stimulated (10^−9 ^M and 10^−6^ M) versus nonstimulated NC PMNLs (no PAF), *n* = 10.

PMNL priming was manifested by increased rates of superoxide release and amplified levels of membrane CD11b [16–18]. Preincubation of isolated normal control (NC) PMNLs with increasing concentrations of PAF caused a dose-dependent increase in the rate of superoxide release from PMA-stimulated PMNLs (^*∗*^ *P* < 0.05, Figure 1(a)). In addition, the expression of CD11b was higher in PAF-treated NC PMNLs compared to nontreated NC PMNLs (^*∗*^ *P* < 0.05, Figure 1(b)).

We reported previously that PMNLs from hemodialysis (HD) patients are primed [15]. To confirm these results, we isolated HD PMNLs and measured their priming. The rate of superoxide release following PMA stimulation was higher in PMNLs isolated from HD patients compared to PAF untreated NC cells (33.5 ± 4 versus 24.7 ± 5 nmoles/10^6^ cells/10 min, resp., ^*∗*^ *P* < 0.05, Figure 1(a)) and was similar to the rate achieved by stimulation with the highest concentration of PAF. The expression of CD11b was also higher in HD PMNLs than in NC PMNLs (61 ± 25 versus 29 ± 11 MFI, resp., ^*∗*^ *P* < 0.05, Figure 1(b)) and comparable to the levels measured in NC cells stimulated with the highest concentration of PAF.

### 3.2. Dose-Dependent Effect of Heparin on PMNL Apoptosis and Priming

**Figure 2:**
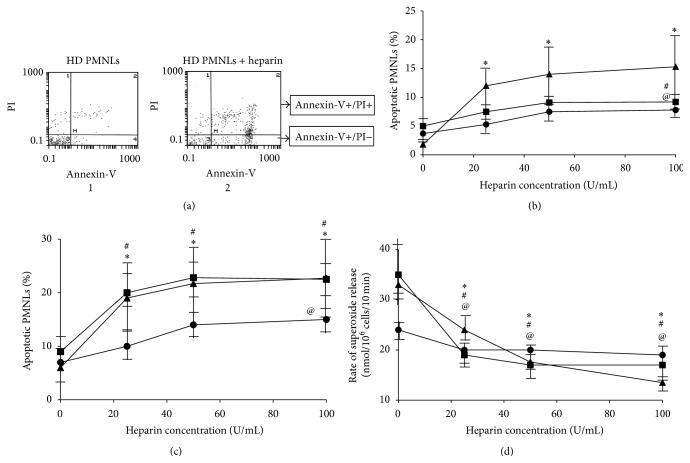
Dose-dependent effect of heparin on PMNL apoptosis and priming. (a) Representative histograms for apoptosis of HD PMNLs without heparin (1) and with 100 U/mL heparin (2) based on Annexin-V-FITC and PI binding. Cell populations: Annexin-V−/PI−, Annexin-V+/PI−, and Annexin-V+/PI+ were regarded as alive, early apoptotic, and late apoptotic or necrotic, respectively. (b) Early apoptosis (Annexin-V+/PI−) in NC PMNLs without stimulation (●) and after 15 min stimulation with 1 *μ*M PAF (■) and HD PMNLs (▲). Cells were preincubated with PAF, washed, and incubated with increasing concentrations of heparin for 30 min. Apoptosis was determined by flow cytometer analysis, using a commercial kit for detection of Annexin-V as described in Section 2. Data are expressed as percentage of apoptotic PMNLs; *n* = 10. ^*∗*^ *P* < 0.05 for HD at 25, 50, and 100 U/mL heparin versus no heparin. ^#^ *P* < 0.05 for NC pretreated with 1 *μ*M PAF incubated with 100 U/mL heparin versus no heparin. ^@^ *P* < 0.05 for NC at 100 U/mL heparin versus no heparin. (c) Early and late (total) apoptosis (sum of Annexin-V+/PI− and Annexin-V+/PI+) in NC PMNLs without stimulation (●) and after 15 min stimulation with 1 *μ*M PAF (■) and HD PMNLs (▲). ^*∗*^ *P* < 0.05 for HD at 25, 50, and 100 U/mL heparin versus no heparin. ^#^ *P* < 0.05 for NC pretreated with 1 *μ*M PAF incubated with 25, 50, and 100 U/mL heparin versus no heparin. ^@^ *P* < 0.05 for NC at 100 U/mL heparin versus no heparin. (d) Superoxide release from NC PMNLs without stimulation (●) and after 15 min stimulation with 1 *μ*M PAF (■) and HD PMNLs (▲). Following preincubation with PAF, cells were washed and incubated with increasing concentrations of heparin for 30 min. The rate of superoxide release was determined after cells activation with 0.32 × 10^−7 ^M PMA. The changes in optical density were monitored at 549 nm continuously in the presence of 0.08 mM cytochrome C. Data are represented by nmoles/10^6^ cells/10 min; *n* = 10. ^*∗*^ *P* < 0.05 for HD at 25, 50, and 100 U/mL heparin versus no heparin. ^#^ *P* < 0.05 for NC pretreated with 1 *μ*M PAF incubated with 25, 50, and 100 U/mL heparin versus no heparin. ^@^ *P* < 0.05 for NC at 25, 50, and 100 U/mL heparin versus no heparin.

We have previously reported that heparin exerts an apoptotic effect on PMNLs [13]. To determine whether primed PMNLs are differently affected by heparin compared to nonprimed cells, we exposed 3 groups of PMNLs: NC, 1 *μ*M PAF-stimulated NC, and HD PMNLs, to increasing concentrations of heparin for 30 min (Figure 2). We used 1 *μ*M PAF-stimulated NC since the rate of superoxide release and the expression of CD11b on these* ex vivo* primed cells were similar to* in vivo* primed cells, isolated from HD patients (Figure 1). Incubation of PMNLs with increasing concentrations of heparin resulted in an increase in early apoptosis in all three groups, however to a much greater extent in HD PMNLs (Figure 2(b)). The increase in HD PMNL apoptosis was significant at all heparin concentrations versus without heparin (*P* < 0.05), while in NC PMNLs and PAF-stimulated NC PMNLs significance was achieved only at 100 U/mL of heparin (*P* < 0.05). Moreover, the maximal percentage of early apoptotic PMNLs was 8% in NC and 10% in PAF-stimulated NC whereas 17% apoptotic cells were detected in HD PMNLs.

Incubation of PMNLs with increasing concentrations of heparin caused a significant increase in apoptosis (early + late) in all three groups of PMNLs, however to a much greater extent in PAF-stimulated NC and HD PMNLs with both groups showing similar dose-dependent responses (Figure 2(c)). The increased degree of apoptosis in PAF-stimulated NC and HD PMNLs was significant at all heparin concentrations versus without heparin (*P* < 0.05), while in NC PMNLs significance was detected only at 100 U/mL of heparin (*P* < 0.05).

The effect of heparin on PAF-stimulated NC and HD PMNLs was much greater than its effect on NC PMNLs (*P* < 0.05 NC versus HD and PAF-stimulated NC at all heparin concentrations). The maximal percentage of apoptotic PMNLs in NC was only 14%, whereas 22% apoptotic cells were detected in PAF-stimulated NC and HD PMNLs.

Priming is also reflected by the rate of superoxide release from PMA-activated PMNLs. To determine whether apoptosis induced by heparin modulates activation of primed PMNLs, we investigated the dose-dependent effect of heparin on the rate of superoxide release. A significant reduction in the rate of superoxide release was observed in all groups of PMNLs (*P* < 0.05 for all heparin concentrations versus no heparin; Figure 2(d)). Moreover, PAF-stimulated NC and HD PMNLs showed lower rates of superoxide release versus NC PMNLs.

The decreased rate of superoxide release was maximized at heparin concentrations of 25 U/mL for NC PMNLs and 50 U/mL for 1 *μ*M PAF-stimulated NC and HD PMNLs. This result suggests that the effect of heparin is greater with increased priming; the maximal inhibition in the rate of superoxide release from primed PMNLs was approximately three times higher than in unprimed cells.

### 3.3. Time-Dependent Effect of Heparin on PMNLs

**Figure 3:**
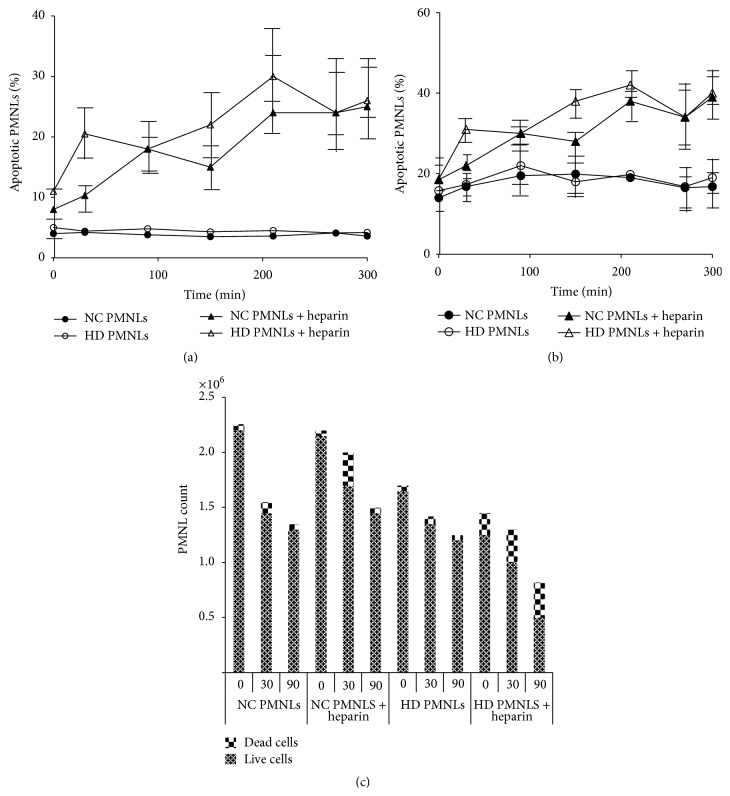
((a), (b)) Early and total apoptosis (resp.) in PMNLs separated from NC (▲) and HD (△) after incubation with 100 U/mL of heparin for 300 min. The results of NC (●) and HD (○) represent the apoptotic levels throughout 300 min without heparin. Apoptosis was determined by flow cytometry using specific FITC-labeled Annexin-V, as described in Section 2. Data are expressed as percentage of apoptotic PMNLs; *n* = 7. (c) PMNL count using trypan blue exclusion assay: NC and HD PMNLs were incubated with 0 and 100 U/mL of heparin for 0, 30, and 90 minutes. Data is expressed as cells counts; *n* = 3.

To determine whether heparin exerts a similar time-dependent apoptotic effect on primed versus quiescent cells, we incubated NC and HD PMNLs with 100 U/mL of heparin up to 300 min (Figure 3). We used 100 U/mL of heparin as it was the only heparin concentration that induced apoptosis in NC PMNLs (Figures 2(b) and 2(c)). Heparin induced early apoptosis in both HD and NC PMNLs at all time points during the incubation, while without heparin, these cells did not exhibit enhanced early apoptosis at any time point (Figure 3(a)). Moreover, heparin caused an oscillatory apoptotic pattern of HD and NC PMNLs reaching apoptotic peaks at 30 and 210 min for HD PMNLs and 90 and 210 min for NC PMNLs. When detecting total apoptosis (early + late), the oscillatory apoptotic patterns of HD and NC PMNLs were similar regarding the apoptotic peaks, but with an augmented percentage of apoptotic cells at all points (Figure 3(b)). To test whether the oscillatory pattern could be explained by the loss of apoptotic cells in the* ex vivo* incubation, we counted the NC and HD PMNLs with and without heparin up to 90 min. This assay revealed that during the 30 min of incubation most of the cells (NC, HD) were still alive, while after 90 min a significant decrease in HD PMNL count was found (Figure 3(c)).

Based on these results, all incubation experiments were for 30 minutes to avoid significant cell disintegration* in vitro*.

### 3.4. The Effect of Priming on Heparin Binding to PMNLs

**Figure 4:**
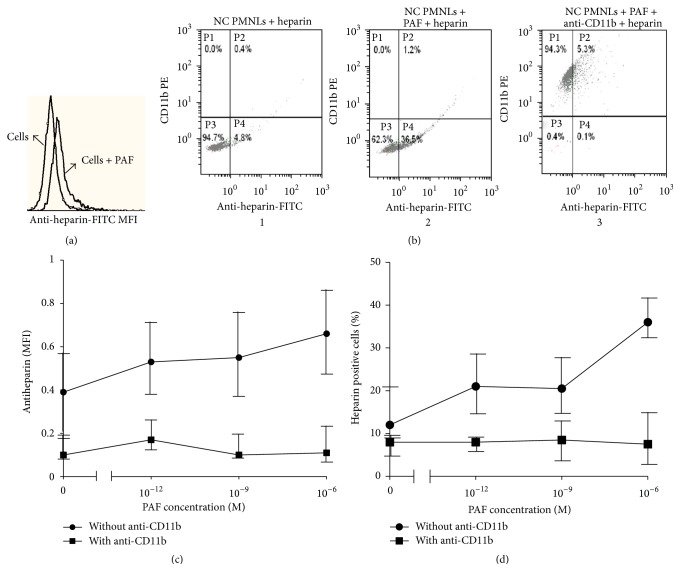
The effect of priming on heparin binding to PMNLs. (a) A representative histogram of flow cytometry, showing heparin binding intensity in NC PMNLs and NC PMNLs stimulated with 10^−6 ^M PAF. (b) Representative histograms of flow cytometry showing percentage of heparin binding cells after incubation with 25 U/mL heparin for 30 min: NC PMNLs without PAF stimulation (1), NC PMNLs with 10^−6 ^M PAF stimulation (2), and NC PMNLs with 10^−6 ^M PAF stimulation and exposure to anti-CD11b-PE antibody (3). ((c), (d)) Levels of heparin bound to NC PMNLs (MFI) and percent of NC PMNLs that bind heparin, respectively, after preincubation with increasing concentration of PAF, determined by flow cytometry using specific FITC-labeled anti-heparin antibody, as described in Section 2 (●). Levels of heparin bound to PMNLs were also measured after preincubation of the cells with anti-CD11b antibodies (■) before the incubation with heparin (*n* = 3).

PAF stimulation resulted in a dose-dependent increase in heparin binding to PMNLs (Figures 4(a)–4(c)). Pretreatment of PAF-stimulated PMNLs with anti-CD11b antibodies completely prevented heparin binding to PMNLs, indicating that heparin binds to PMNLs, at least partially, via CD11b (Figure 4(c)). These results were confirmed by additional studies using heparin that was conjugated to FITC. Heparin binding intensity to unstimulated NC PMNLs was 6.22 ± 0.19 MFI and increased to 13.15 ± 1.76 MFI after stimulation with 1 *μ*M PAF, demonstrating increased heparin binding with increased priming. Pretreatment of PAF-stimulated PMNLs with anti-CD11b antibodies completely prevented heparin binding to PMNLs with heparin-FITC intensity reduced to 6.48 ± 0.57 MFI.

In a second set of experiments depicted in Figure 4(d), FITC-conjugated anti-heparin antibodies were used to detect the percentage of PAF-stimulated PMNLs which bound heparin. The more primed the PMNLs, the higher the percentage of cells that bound heparin. This binding was also prevented by preincubation with anti-CD11b antibodies applied before the addition of heparin (Figure 4(d)). These results indicate that heparin binding is mediated by CD11b and that the higher the priming state is, the more the heparin binds to PMNLs.

### 3.5. The Effect of Priming on Apoptosis Induced by Heparin

**Figure 5:**
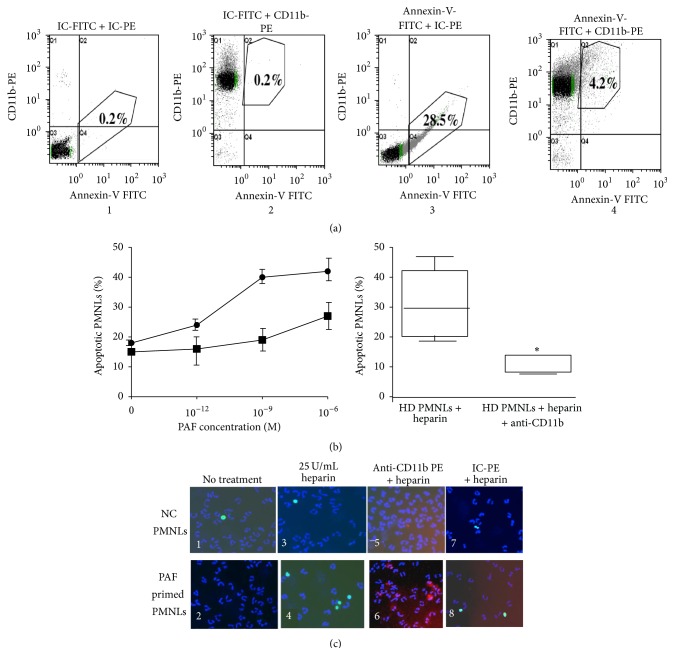
The effect of priming on apoptosis induced by heparin. (a) Representative histograms of flow cytometry showing the percentage of apoptotic cells after incubation with 25 U/mL heparin for 30 min: HD PMNLs with IC-FITC and IC-PE (1), HD PMNLs with IC-FITC and anti-CD11b-PE antibody (2), HD PMNLs with Annexin-V-FITC and IC-PE (3), and HD PMNLs with Annexin-V-FITC and anti-CD11b-PE antibody (4). (b) Apoptosis in NC PMNLs (●) after 15 min stimulation with increasing concentrations of PAF. Cells were preincubated with PAF, washed, and subjected to 30 min incubation with 25 U/mL heparin. Apoptosis was determined by flow cytometer analysis, using Annexin-V commercial kit as described in Section 2. Data are expressed as percentage of apoptotic PMNLs; *n* = 3. In addition, the percentage of apoptotic NC PMNLs that bind heparin was also measured after preincubation of NC separated PMNLs with PAF and anti-CD11b antibodies (■), before heparin incubation. The boxes and whiskers presentations represent apoptosis in HD PMNLs incubated with 25 U/mL heparin for 30 min, with and without incubation with anti-CD11b antibodies. (c) Apoptosis in NC PMNLs (upper left panel 1) and 15 min after stimulation with 10^−6 ^M PAF (lower left panel 2). These cells were treated with 25 U/mL heparin for 30 min (panels 3 and 4). Apoptosis was evaluated by the TUNEL method (FITC green staining). Nuclei were stained with Hoechst reagent (blue staining). The percent of apoptotic NC PMNLs that bind heparin was measured after preincubation of the PMNLs with anti-CD11b antibodies (red staining) (panels 5 and 6) or IC-PE antibodies (panels 7 and 8) before incubation with heparin. Quantification of apoptosis: over 300 cells were counted, from at least three independent experiments.

PAF-stimulated NC PMNLs showed increased apoptosis dependent on the cell priming state when incubated with heparin (Figures 5(a) and 5(b)) and apoptosis was increased concomitantly with increases in cell priming. Anti-CD11b which prevents the binding of heparin to PMNLs abolished the apoptotic effect of heparin at all PAF concentrations (Figure 5(b)). In addition, anti-CD11b also prevented the binding of heparin to primed PMNLs isolated from HD patients and almost completely abolished the apoptosis induced by heparin (Figure 5(b)).

The effect of 25 U/mL heparin on apoptosis of NC PMNLs was also examined before and following the stimulation with 1 *μ*M PAF, using the TUNEL assay. Representative results are shown in Figure 5(c). Very few apoptotic cells (with green nuclei, ~2%) were observed in NC PMNLs (Figure 5(c)(1)) and NC PMNLs stimulated by PAF (Figure 5(c)(2)) without exposure to heparin. When NC PMNLs were exposed to 25 U/mL heparin, the percentage of apoptotic cells increased to 6 ± 2%, together with 25% cell loss (Figure 5(c)(3)). When PAF-stimulated NC PMNLs were exposed to 25 U/mL heparin, the percentage of apoptotic cells increased to approximately 22 ± 5% (Figure 5(c)(4)), concomitantly with 65% cell disappearance. Exposure of the cells to anti-CD11b-PE antibodies prior to the addition of heparin completely abolished the apoptotic effects of heparin and cell disappearance (Figures 5(c)(5) and 5(c)(6)) on both NC PMNLs and NC PMNLs stimulated by PAF. Under the same conditions, exposure to isotype-controls- (IC-) PE (instead of to anti-CD11b-PE) did not prevent the apoptotic effects of heparin and cell disappearance (Figures 5(c)(7) and 5(c)(8)). These results clearly demonstrate that heparin-mediated apoptosis depends on PMNL priming.

**Figure 6:**
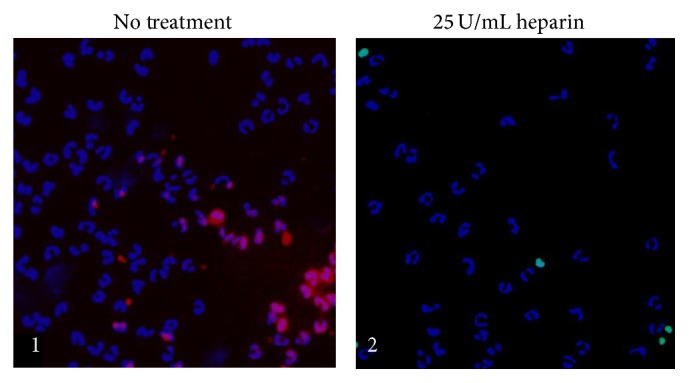
CD11b expression on primed PMNLs. CD11b expression on NC PMNLs after 15 min stimulation with 10^−6 ^M PAF without an additional treatment (1) and with 30 min incubation with 25 U/mL heparin (2). Apoptosis was evaluated by the TUNEL method (FITC green staining). Nuclei were stained with Hoechst reagent (blue staining).

In order to detect whether exposure of the cells to heparin causes loss of primed PMNLs, we further stained the PAF-primed PMNLs with anti-CD11b-PE (Figure 6). Thirty percent of the PAF-treated cells that were not exposed to heparin showed elevated levels of CD11b, supporting their primed state. When these cells were exposed to heparin, no primed PMNLs or decreases in cell count could be seen.

### 3.6. The Effect of Anti-CD11b on Superoxide Release in the Presence of Heparin

**Figure 7:**
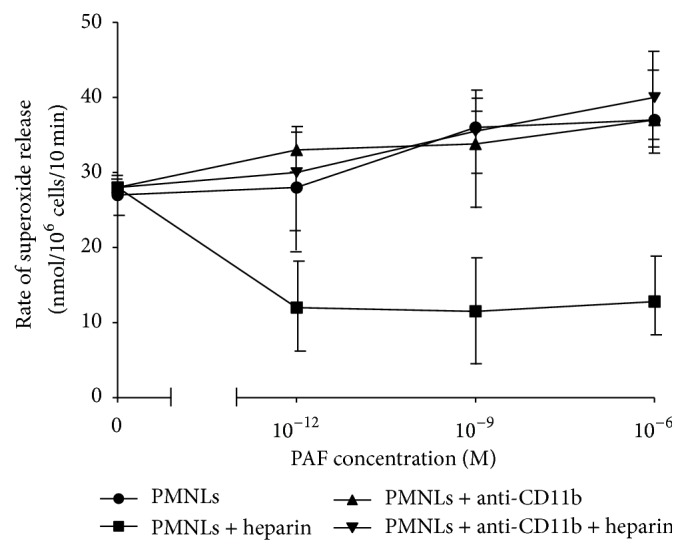
The effect of anti-CD11b on superoxide release in the presence of heparin. Superoxide release from NC PMNLs preincubated with increasing concentrations of PAF and IC-PE antibodies (●). In addition, superoxide release from NC PMNLs was also measured after 15 min preincubation with PAF and anti-CD11b-PE antibodies (▲). Furthermore, cells were washed and subjected to 30 min incubation with 25 U/mL of heparin (■). The rate of superoxide release from NC PMNLs was also measured after preincubation of NC PMNLs with PAF + anti-CD11b antibodies, before heparin incubation (▼). The rate of superoxide release was determined with 0.32 × 10^−7^ M PMA-stimulated PMNLs. The changes in optical density were monitored at 549 nm continuously in the presence of 0.08 mM cytochrome C. Data are represented as nmoles/10^6^ cells/10 min; *n* = 3.

Since heparin causes a decrease in superoxide release (Figure 2(d)) and at the same time it induces apoptosis via CD11b, it was interesting to find out whether blocking CD11b would have an effect on superoxide release. PAF-stimulated NC PMNLs incubated with IC showed increased rates of superoxide release which were dependent on the extent of the cell priming: the greater the priming state, the greater the rate of superoxide release (PMNLs; Figure 7). Heparin (25 U/mL) prevented this increase in superoxide release (PMNLs + heparin; Figure 7). PAF-stimulated NC PMNLs, incubated with anti-CD11b but not with heparin, showed a similar increased rate of superoxide release as with IC, which was also dependent on the extent of the cell priming (PMNLs + anti-CD11b; Figure 7). When anti-CD11b was added to PMNLs prior to the incubation of the cells with heparin (PMNLs + anti-CD11b + heparin), the effect of heparin in terms of inhibition of superoxide release was abolished (Figure 7).

## 4. Discussion

In this study, we examined the effect of heparin on PMNLs. Our novel finding is that heparin exerts its apoptotic effect on primed PMNLs by binding to their CD11b. Moreover, heparin causes a significant dose-dependent decrease in the rate of superoxide release from PMNLs, blocking primed cells from further activation, partially explaining the anti-inflammatory effects of heparin.

It has been previously reported that heparin induces apoptosis in a dose-dependent manner in NC PMNLs [13]. In the present study, 30 min incubation with heparin enhanced apoptosis in separated human PMNLs in a dose-dependent fashion, however to a much greater extent in primed PAF-stimulated NC and HD PMNLs. In NC PMNLs, the increase in apoptosis was mild and became significant only at 100 U/mL of heparin. Interestingly, apoptosis time courses for NC or HD PMNLs incubated with heparin displayed an oscillatory pattern, with NC apoptosis lagging that of the HD PMNLs. This oscillatory behavior can be explained by the fact that 100 U/mL of heparin caused a time-dependent increase in apoptosis both in NC and in HD PMNLs (however faster and to a much greater extent in HD). The decline in apoptosis, for example, after 90 min for HD PMNLs and only after 150 min for NC PMNLs, can be explained by disappearance of the apoptotic cells as shown in our* ex vivo* survival studies: almost 40–50% of the HD PMNLs disappeared after 90 min, probably by disintegration. Why do we see another increase after the nadirs? We suggest that the next apoptotic peak is the result of an increase in CD11b expression* in vitro *during incubation in PBS (data not shown), which in the presence of heparin will result in apoptosis. Nevertheless, in the absence of heparin, such an increase in CD11b is not accompanied by enhanced apoptosis. Another possible explanation for increased apoptosis after 30 minutes can arise from a toxic effect. While we specifically measured priming and apoptosis, we do recognize that cell counts were decreasing over time (an effect we referred to as cell disintegration), which may suggest toxicity. Yet, when measuring cell activation, we made sure to count the same number of cells for each time point. Moreover, other markers of activation and priming were measured only on viable cells. Thus, even if cell toxicity is possible due to prolonged activation, the rate of superoxide release and levels of CD11b were measured on viable cells and support our conclusion that heparin can increase apoptosis through its actions mediated by CD11b.

Increases in PMNL priming as expressed by increased levels of CD11b and superoxide release resulted in dose-dependent increases of heparin binding to PMNLs. This increased binding is demonstrated both by the augmented intensity of heparin bound to the PMNLs and by the percentage of cells binding anti-heparin antibodies following incubation with heparin. In all experiments anti-CD11b antibodies competed with heparin for binding to CD11b, supporting the proposed mechanism of heparin-CD11b interactions as previously reported [11, 12]. Moreover, the percentage of PMNLs that bind heparin depends on the initial priming state of the cells; that is, a higher priming state results in a higher percentage of cells binding heparin. Finally, increases in cell priming resulted in augmented apoptosis by heparin in PMNLs, which was abolished by anti-CD11b antibodies added prior to the addition of heparin.

Heparin inhibited superoxide release from PMNLs and to a greater extent when cells were primed, such as HD and PAF-stimulated NC PMNLs. This indicates that heparin exerts its effect mainly on the subpopulation of cells that are primed and are more abundant in HD and PAF-stimulated PMNLs. Since the population of primed PMNLs is the major contributor to superoxide release, rendering them apoptotic by heparin results in a decreased rate of superoxide release. These important findings indicate that the rates of superoxide release are increased due to a primed subpopulation of cells. In addition, the inhibitory effect of heparin on superoxide release from PMNLs was also abolished by anti-CD11b antibodies added prior to the addition of heparin.

Altogether, this study suggests a novel role for the interaction of heparin and PMNL CD11b, resulting in cell apoptosis. Apoptosis induced by heparin occurs when CD11b levels are increased or, in other words, when a subpopulation of primed PMNLs exists. As a result, heparin plays an anti-inflammatory role by making the primed cells apoptotic and preventing leakage of PMNL contents into the surrounding milieu. Heparan sulfate, a less sulfated form of heparin, is also known to bind to the PMNL CD11b [11], and recently we found it can promote apoptosis in separated PMNLs (data not shown), a characteristic that is dose-dependently affecting superoxide release from stimulated PMNLs [22]. The higher the heparan sulfate concentration, the lower the rate of superoxide release. More interesting, however, is the* in vivo* presence of heparan sulfate in the ECM and on the endothelial cell surface of vascular walls. Our studies on heparin raise the intriguing possibility that heparan sulfate may limit and/or regulate, to a certain degree, tissue injury and vascular damage by uncontrolled degranulation and release of reactive oxygen species and toxic contents into the surrounding milieu by activated transmigrating PMNLs.
